# Predictors of HIV Testing among Patients with Tuberculosis in North West Ethiopia: A Case-Control Study

**DOI:** 10.1371/journal.pone.0009702

**Published:** 2010-03-15

**Authors:** Animut Ayenew, Abenet Leykun, Robert Colebunders, Amare Deribew

**Affiliations:** 1 Federal Ministry of Health, Addis Ababa, Ethiopia; 2 Private Sector Partnership -Ethiopia, Addis Ababa, Ethiopia; 3 Department of Clinical Sciences, Institute of Tropical Medicine, Antwerp, Belgium; 4 Department of Epidemiology and Social Medicine, University of Antwerp, Antwerp, Belgium; 5 Department of Epidemiology, Jimma University, Jimma, Ethiopia; University of Cape Town, South Africa

## Abstract

**Background:**

The acceptance of HIV testing among patients with tuberculosis (TB) is low in Ethiopia. The purpose of this study was to assess predictors of acceptance of HIV testing among patients with TB in North Ethiopia.

**Methods:**

A case control study was conducted in eight randomly selected health facilities in North Ethiopia from February 5 to March 11, 2009. A total of 282 participants (188 controls and 94 cases) were included in the study. Cases were TB patients who refused to be tested for HIV. We used quantitative and qualitative methods of data collection. For the quantitative survey, cases and controls were interviewed by trained nurses using a pre-tested and structured questionnaire. In-depth interviews were conducted with 5 nurse counselors and 15 TB patients. Bivariate and multivariate analysis was done using SPSS 16.0 statistical software.

**Results:**

The uptake of HIV testing among TB patients in the study health facilities was 70.6%. The rate of TB/HIV co-infection in those who were tested was 36.2%. From the source population, a total of 282 participants were included in the study. TB patients who had formal education [OR = 2.35, (95%CI: 1.33, 4.13)], high awareness about the benefits of HIV counseling and testing [OR = 3.14, 95%CI: 1.77, 5.50)], and a low stigmatized attitude [OR = 3.16, 95%CI: 1.79, 5.59)] were more likely to accept HIV testing. The qualitative study also revealed that low awareness and stigma were the major reasons for non-acceptance of HIV testing.

**Conclusion:**

“Knowledge and attitude” factors were the major barriers for HIV testing. Tailored training should be given to TB patients and the community concerning the benefits of HIV testing. During counseling sessions, health workers should focus on barriers of uptake of HIV testing such as stigma and discrimination.

## Introduction

Globally, there are 1.4 million HIV positive tuberculosis (TB) cases [1]. In Ethiopia, TB is the leading cause of morbidity, one of the three major causes for hospital admission, and the second killer next to malaria [2]. The rate of TB/HIV co-infection in Ethiopia ranges from 40–70% [3]–[5].

Collaborative TB/HIV activities are essential to reduce TB related mortality among patients with HIV infection [6]. Provider initiated counseling and testing (PICT) for patients with TB is one of the key strategies of TB/HIV collaborative activities and offers an entry point for HIV/AIDS care [6]–[7]. Ethiopia incorporated PICT in its national TB/HIV policy in 2005 and the service is being expanded to the level of health centers in different parts of the country [2].

In spite of the rapid expansion of PICT in Ethiopia, studies revealed that the uptake of HIV testing among TB patients [8] was low compared to other studies in Africa and Asia [9]–[13]. Little is known about the reasons for this low acceptance rate of HIV testing by TB patients in Ethiopia. The aim of this case control study was to assess predictors of HIV testing among TB patients in North West Ethiopia.

## Materials and Methods

We conducted a case control study from February 5 to March 11, 2009 in East Gojjam, North Ethiopia. The East Gojjam zone is divided into 18 administrative Districts. The zone has one zonal hospital, one district hospital, 18 health centers, 384 health posts, and 51 private clinics. The two hospitals and all health centers provide HIV counseling and Testing (HCT) and Directly Observed, Short courses Therapy (DOTS). We randomly selected 7 health centers and 1 hospital.

The study population consisted of cases and controls who had regular follow up for one year prior to the survey in the TB/HIV clinics of the selected health facilities. Cases were TB patients who refused HIV testing and controls were TB patients who accepted HIV testing. The diagnosis of TB was based on the national guideline [2]. HIV testing was done according to the national protocol [14] using the KHB test (Shanghi Kehua Bio-engineering, Ltd, 2008, China). The sample size was calculated using Epi Info 6.04 statistical software (Center for Disease Control and Prevention, Atlanta, 2005). The following parameters were used to calculate the sample size: a proportion of literacy among cases and controls of 73% and 88.2% respectively [15], CI of 95%, a power of 80% and a case to control ratio of 1:2, and 10% for non-response. Among several predictor variables, literacy status was selected since it required the largest sample size. The total sample size was 282 (94 cases and 188 controls). Cases and controls were identified from the registration books of the TB clinics. For each case, two controls were selected by simple random sampling method using the patients' unique identification number. TB patients less than 15 years and who discontinued their treatment were excluded from the study.

We used quantitative and qualitative methods of data collection. For the quantitative survey, cases and controls were interviewed by trained nurses using a pre-tested and structured questionnaire which was adapted from published questionnaires [16], [17]. The questionnaire was prepared first in English and translated into Amharic (local language) and retranslated back to English to check for consistency. The questionnaire consisted of socio- demographic variables; knowledge on HIV, counseling and testing and TB; history of TB treatment; perception on stigma towards people living with HIV; perceived personal risk for HIV; awareness about the benefits and barriers of HIV testing. Perceived personal risk for HIV was categorized as “no risk”, “low”, “moderate”, or “high” risk. Knowledge on TB/HIV was measured by asking questions on mode of transmission, means of prevention, and misconception about TB/HIV. A correct answer was given a score 1 and an incorrect answer a score 0. The sum was computed and those who scored above the mean were labeled as knowledgeable. Perceived stigma of the study participants towards people living with HIV/AIDS was assessed using 12 questions with “yes/no” responses. The stigma questions focused on the effect of HIV/AIDS on marriage, relationship with family and society, physical or sexual abuse and emotional support by family and friends. A score of 1 and 0 of the questions indicated presence and absence of perceived stigma respectively. A mean score was computed and respondents who scored above the mean were labeled as having a high stigmatized attitude. Five questions were asked about the benefits of HIV testing. Awareness about the benefits of HIV testing was assessed based on the mean score of the items. Individuals who scored above the mean were labeled as having high awareness about the benefits HIV testing.

For the qualitative survey, we conducted in-depth interview with 5 nurse counselors and 15 TB patients using interview guides. The major focus of the in-depth interview was to find out major barriers for HIV testing.

Data were cleaned for inconsistencies and missing value and analyzed using SPSS 16.0 statistical software. Pearson's correlation was used to evaluate the association between independent variables and uptake of HIV testing. To control for the effect of confounding variables, a stepwise logistic regression was done. Variables with a significant association in bivariate analysis were included in a final logistic regression model. Qualitative data were categorized using key thematic areas and the data was interpreted and presented using verbatim.

Ethical clearance was obtained from the ethical committee of Jimma University. Written consent was obtained from the study participants and confidentiality was assured for all the information provided.

## Results

A total of 333 TB patients attended the study health facilities during the study period. The uptake of HIV testing among these patients was 70.6%. The rate of TB/HIV co-infection in the source population was 36.2% (**Table 1**).

**Table 1:** Uptake of HIV testing and HIV/TB co-infection, Northwest Ethiopia.

Name of Health Facility	Total number of TB patients	Accepted HIV testing No (%)	HIV/TB co-infection No (%)
Debre Markos Health center	110	90(81.8)	26(28.9)
Lumame health center	37	14(37.8)	6(42.8)
Dejen health center	21	15(71.4)	5(33.3)
Bichena health center	25	18(72.0)	6(33.3)
Debrwork health center	67	51(76.1)	24 (47.1)
Motta hospital	21	14(66.7)	6(42.8)
Kuy health center	25	14(56.0)	5(35.7)
Amanuel health center	27	19(70.4)	7(36.8)
Total	333	235(70.6)	85(36.2)

A total of 282 study participants were selected from the source population. Of the total study participants, 188 were controls (tested for HIV) and 94 were cases (refused to be tested for HIV). Among the controls, 19(10.1%) were tested for HIV before the diagnosis of TB. The remaining 169(89.9%) were tested during TB treatment.

Of the total study population, 154(54.6%), 67(23.8%), and 61(21.6%) of them had smear negative, smear positive and extra-pulmonary TB respectively.

There were no differences in age, sex and marital status between cases and controls. In contrast, in the bivariate analysis, individuals who had formal education and government employees were more likely to have been tested for HIV (**Table 2**).

**Table 2:** Association of socio-demographic factors and acceptance of HIV testing among TB patients in East Gojjam, North West Ethiopia.

Variables	Controls (n = 188)	Cases(n = 94)	P-value
**Age(years)**			0.1
15–24	53(28.2)	34(36.2)	
25–34	77(40.1)	27(28.7)	
> = 35	58(30.9)	33(35.1)	
**Sex**			0.5
Male	111(59)	59(62.8)	
Female	77(41)	35(37.2)	
**Residence**			0.2
Rural	85(45.2)	50(53.2)	
Urban	103(54.8)	44(46.8)	
**Marital status**			0.4
Married	92(48.9)	52(55.3)	
Single	53(28.2)	28(29.8)	
Divorced	33(17.6)	8(8.5)	
Widowed	10(5.3)	6(6.4)	
**Formal Education**			0.001
Yes	134(71.3)	43(45.7)	
No	54(28.7)	51(54.3)	
**Occupation**			0.001
Government employee	47(25.0)	5(5.3)	
Merchant	18(9.6)	14(14.9)	
Farmer	36(19.1)	25(26.6)	
House wife	12(6.4)	13(13.8)	
Daily laborer	34(18.1)	10(10.6)	
Student	28(14.9)	16(17.1)	
Jobless	13(6.9)	11(11.7)	

The following variables: knowledge on HIV/TB (P = 0.001), ever heard of provider initiated HIV counseling and testing (P = 0.001), perceived risk of HIV infection (P = 0.001), awareness of the benefits of HIV testing (P = 0.001), and perceived stigma (P = 0.001) were strongly associated with uptake of HIV testing (**Table 3**).

**Table 3:** Association of knowledge and attitude factors and acceptance of HIV testing among TB patients in East Gojjam, Northwest Ethiopia.

Variables	Controls (n = 188)	Cases(n = 94)	P-value
**Knowledge on TB/HIV**			0.001
Non-knowledgeable	74(39.4)	57(60.6)	
Knowledgeable	114(60.6)	37(39.4)	
**Perception on the risk of HIV infection**			0.001
Low	111(59.0)	82(87.2)	
High	77(41.0)	12(12.8)	
**Ever heard about provider initiated HIV testing**			0.001
Yes	166(88.3)	68(72.3)	
No	22(11.7)	26(27.2)	
**Perceived benefit of HIV testing**			0.001
High	126(67.0)	38(40.4)	
Low	62(33.0)	56(59.6)	
**Disclosure of the TB**			0.16
Yes	170(90.4)	80(85.1)	
No	18(9.6)	14(14.9)	
**Perceived stigma**			0.001
Low	116(61.7)	29(30.9)	
High	72(38.3)	65(69.1)	

In multivariate analysis, individuals who had formal education were 2.35 times more likely to be tested for HIV than illiterates, [OR = 2.35, (95%CI: 1.33, 4.13)]. Individuals with a high awareness about the benefits of HIV counseling and testing were more likely to be tested than individuals with low awareness, [OR = 3.14, 95%CI: 1.77, 5.50)]. TB patients who had a low stigmatized attitude [OR = 3.16, 95%CI: 1.79, 5.59)] were 3.2 times more likely to be tested than individuals who had high stigmatized attitude (**Table 4**).

**Table 4:** Factors independently associated with HIV testing among TB patents in East Gojjam, North West Ethiopia.

Variables	Controls (n = 188)	Cases(n = 94)	Crude OR (95% CI)	Adjusted OR (95%CI)
**Formal Education**				
Yes	134(71.3)	43(45.7)	2.94(1.76, 4.9)	2.35(1.33, 4.13)
No	54(28.7)	51(54.3)	1.00	1
**Ever heard about provider initiated HIV testing**				
Yes	166(88.3)	68(72.3)	2.88(1.53, 5.44)	3.43(1.67,7.0)
No	22(11.7)	26(27.2)	1	1
**Perceived benefit of HIV testing**				
High	126(67.0)	38(40.4)	2.85(1.79, 4.99)	3.14(1.77, 5.50)
Low	62(33.0)	56(59.6)	1	1
**Perceived stigma**				
Low	116(61.7)	29(30.9)	3.61(2.13, 6.12)	3.16(1.79,5.59)
High	72(38.3)	65(69.1)	1.00	1

Qualitative finding also revealed that low awareness about the association between TB and HIV, stigma and discrimination of people who have TB and live with HIV, low perceived risk and partners trust were the major reasons for non-acceptance of HIV testing. One third of the TB patients believed that TB and HIV have no any relationship. All of the interviewed counselors and TB patients pointed out that stigma towards TB and HIV patients is widespread in the community and many of the TB patients refused to be tested because of fear for a positive test result and the associated stigma (**Table 5**).

**Table 5:** Examples of interview extracts of TB patients and counselor concerning barriers for HIV testing.

Barriers for HIV testing	Patient and counselors citations
Lack of knowledge concerning the association between TB and HIV	*“TB is an ancient disease that exists before HIV; I don't want to be tested since the two diseases have no any association.”* (A 29 years old TB patient, who refused to be tested for HIV)
Discrimination	*“TB patients are highly stigmatized by the community like people living with HIV. They don't want to disclose they have TB and refuse to be tested fearing a positive HIV result and the associated stigma.”* (A 31 years male counselor in Debremarkos health center) *“I personally prefer not to disclose my illness and not to be tested for HIV. If my result is positive, I may lose my relationship with my family and neighbours.”* (A 27 years of TB patient Bichen health center)
Low risk perception	*“Some people refused to be tested since they considered themselves not at risk of contracting HIV.”* (A 23 years male TB patient)
Partners trust	*“I am married and a mother of two children. Since I trust my husband, I don't want to be tested for HIV now.”* (a 36 year old female TB patient)

## Discussion

Early identification of HIV in patients with TB through the different modalities of counseling and testing is one of the key strategies to achieve the TB-related millennium development goals [18]. Several studies in Africa showed that the acceptance of HIV testing among TB patients ranged from 85% to 95% [9]–[12]. Studies done in Indonesia [13] and Thailand [19] indicated that 77% and 93% of TB patients accepted HIV testing respectively. In our study, nearly one third of TB patients declined provider initiated HIV testing. Studies done among TB suspects [unpublished data by the same authors] and TB patients [8] in other parts of Ethiopia showed similar findings. This indicates that the target to reach the recommended 80% HIV testing rate among TB patients in Ethiopia is not yet achieved [20]. In our study, we tried to document determinants of uptake of HIV testing using quantitative and qualitative methods. Education was positively associated with acceptance of HIV testing which is contrary to the study done in south Ethiopia [8] but similar to the finding of Worku et al in pregnant women in Ethiopia [21]. Factors such as knowledge about HIV and provider initiated counselling and testing, perceived benefits of counselling and testing, and perceived stigma were major predictors of acceptance of HIV testing in our study. Demisse et al [22] in east Ethiopia and Worku et al [21] in Addis Ababa did identify that knowledge and perceptions about the benefits of HIV counselling were the major predictors of HIV testing among pregnant women. A study in Indonesia showed that TB patients who had high perception on the benefits of HIV testing were more likely to accept HIV testing [23]. People living with HIV/AIDS have been stigmatized worldwide leading to discrimination and isolation from the society [24]. Mahendradhata et al in Indonesia [23] reported that stigma was the major influencing factor for the non-acceptance of HIV testing among patients with TB. In our study, we found that stigma towards patient with TB and HIV was widespread in the community and was a major reason for non-acceptance of HIV testing. The deep rooted stigma in our study area could have emerged due to different reasons. In the early period of the HIV epidemic in Ethiopia, many of the educational materials contained pictures of emaciated persons with AIDS. These types of educational messages and the lack of community education in rural settings might have created fears about the disease which resulted in deep rooted stigma towards people living with HIV.

Although we have identified several predictors of HIV counseling and testing using both qualitative and quantitative methods, our study has some limitations. Although the stigma and knowledge questionnaire was pretested, the instrument was not validated. Moreover, the use of nurses in the study health institutions as data collectors might have introduced an interviewer bias. Finally, the number of in depth interviews that were carried out was small.

In conclusion, level of education, perceived benefits of HIV testing, knowledge of provider initiated HIV testing and stigma were independently associated with acceptance of HIV testing. To increase the uptake of HIV testing, tailored training of TB patients and the community concerning the benefits of HIV testing, stigma and discrimination should be given using appropriate channels. During counseling sessions, health workers should focus on the aforementioned major barriers for the uptake of HIV testing.

## References

[1] World Health Organization (2009). Global Tuberculosis control: epidemiology, strategy and financing.. WHO.

[2] Federal Democratic Republic of Ethiopia, Ministry of Health (2008). TB/HIV implementation guideline, Addis Ababa, Ethiopia..

[3] Nigussu N, Deribew A, Kassahun W, Ludwig A, Colebunders R (2009). TB/HIV co-infection among suspects of tuberculosis in HIV prevalent setting, Addis Ababa, Ethiopia. Master thesis in public health.. Jimma University.

[4] Kassu A, Mengistu G, Ayele B (2007). Co-infection and clinical manifestation of TB in HIV infected and uninfected adults at teaching hospital, northwest Ethiopia.. J Microbial Immunol Infect.

[5] Demissie M, Lindtjon B, Tegbaru B (2000). Human Immunodeficiency virus (HIV) infection in tuberculosis patients in Addis Ababa.. Ethiop J Health Dev.

[6] WHO/UNAIDS (2004). UNAIDS/WHO policy statement on HIV testing. WHO/HTM/TB/2004.330.. http://www.who.int/hiv/topics/tb/actions/en/.

[7] WHO, UNAIDS and UNICEF (2009). Towards universal access: Scaling up priority HIV/AIDS interventions in the health sector: 2009 Progress Report.. http://www.who.int/hiv/pub/2009progressreport/en/.

[8] Jerene D, Aschalew E, Bernt L (2007). Acceptability of HIV counseling and testing among tuberculosis patients in south Ethiopia.. BMC International Health and Human Rights.

[9] Munthali L, Mwaungulu JN, Munthali K, Bowie C, Crampin A (2006). Using tuberculosis suspects to identify patients eligible for antiretroviral treatment.. Int J Tuberc Lung Dis.

[10] Odhiambo J, Kizito W, Njoroge A, Wambua N, Nganga L (2008). Provider-initiated HIV testing and counseling for TB patients and suspects in Nairobi, Kenya.. Int J Tuberc Lung Dis.

[11] Van Rie A, Sabue M, Jarrett N, Westreich D, Bhets D (2008). evaluation of three implementation models in Kinshasa, Congo.. Int J Tuberc Lung Dis.

[12] Srikantiah P, Lin R, Walusimbi M, Okwera A, Luzze H (2007). Elevated HIV sero-prevalence and risk behavior among Ugandan TB suspects: implications for HIV testing and prevention.. Int J Tuberc Lung Dis.

[13] Mahendradhata Y, Ahmad R, Kusuma T, Boelaert M, Werf V (2008). Voluntary counseling and testing and uptake and HIV prevalence among Tuberculosis patient in Jogjakarta, Indonesia.. Royal Society of Tropical Medicine and Hygiene.

[14] Federal Democratic Republic of Ethiopia, Ministry of Health (2008). Guidelines for laboratory HIV testing in blood safety, surveillance, VCT and ARV use.. Addis Ababa, Ethiopia.

[15] Maru M (2007). Assessment of VCT utilization and willingness to accept provider-Initiated HIV counseling and testing among tuberculosis patients in Addis Ababa.. Master thesis, Addis Ababa University.

[16] Aklilu A (2004). Assessment of Willingness of TB Patients towards VCT in Selected Health Centers, North Gondar Administrative Zone.. MPH Thesis, Addis Ababa University.

[17] Central statistical agency [Ethiopia] and ORC Macro (2006). Ethiopia Demographic and Health Survey, Addis Ababa, Ethiopia and Calverton, Maryland, USA..

[18] WHO (2006). The STOP TB STRATEGY: building on and enhancing the DOTS to meet the TB-related millennium development goals.. http://www.who.int/tb/strategy/en/.

[19] Nateniyom S, Jittimanee SX, Viriyaktjar D, Jittimanee S, Keophaithool, S, et al (2008). Provider initiated diagnostic HIV counseling and testing in tuberculosis clinics in Thailand.. Int J Tuberc Lung Dis.

[20] World Health Organization (2009). Global Tuberculosis Control: Surveillance, Planning, Financing.. WHO report.

[21] Worku G, Enquselassie F (2007). Factors determining acceptance of voluntary HIV counseling and testing among pregnant women attending antenatal clinic at army hospitals in Addis Ababa.. Ethiop Med J.

[22] Demisse A, Deribew A, Abera M (2009). Determinants of acceptance of voluntary HIV testing among antenatal clinic attendees at Dil Chora Hospital, Dire Dawa, East Ethiopia.. Ethiop J Health Dev.

[23] Mahendradhata Y, Ahmad R, Lefèvre P, Boelaert M, Van der Stuyft P (2008). Barriers for introducing HIV testing among tuberculosis patients in Jogjakarta, Indonesia: a qualitative study.. BMC Public Health.

[24] Mawar N, Sahay S, Pandit A, Mahajan U (2005). The Third phase HIV pandemic: Social consequences of HIV/AIDS stigma & discrimination & future needs.. Indian J Med. Research.

